# Vygotsky’s, Leontiev’s and Engeström’s Cultural-Historical (Activity) Theories: Overview, Clarifications and Implications

**DOI:** 10.1007/s12124-022-09703-6

**Published:** 2022-06-10

**Authors:** Ngo Cong-Lem

**Affiliations:** 1 Faculty of Education, Monash University, 19 Ancora Imparo, Clayton, 3800 Victoria Australia; 2 Faculty of Foreign Languages, University of Dalat, Dalat, Vietnam

**Keywords:** Cultural-historical theory, Activity theory, Vygotsky, Education

## Abstract

At the social turn in education, Vygotsky’s cultural-historical/sociocultural theory (VST) has become particularly influential. There are other cultural-historical traditions associated with VST, including Leontiev’s and Engeström’s versions of cultural-historical activity theory (CHAT). These approaches are frequently conflated, resulting in confusion that can be consequential in interpreting educational research findings. Unravelling these frameworks is thus an important and urgent task. In addressing this gap, the paper first provides an overview of the origins and fundamental tenets of these cultural-historical perspectives, followed by a critical evaluation of and comparison among them. Implications for utilising these cultural-historical traditions are discussed.

## Introduction

The sociocultural turn (Block, 2003) has witnessed the dominance of sociocultural theories in education, one of which is Vygotsky’s cultural-historical/sociocultural theory (VST). Lantolf et al. (2021) observe that since its introduction, “the amount of research that has been published within the SCT [i.e. VST as in this paper] framework has grown exponentially” (p. 327). For instance, VST’s concept of zone of proximal development (ZPD) has productively informed teaching practices involving peer learning, scaffolding feedback, dynamic assessment and professional learning community (Aljaafreh & Lantolf, 1994; Lave & Wenger, 1991; Rassaei, 2019).

In addition to VST, cultural-historical activity theory (CHAT) has also become prevalent in research on education. CHAT is often associated with VST, claiming the latter as its first generation (Engeström, 2001). In previous literature, CHAT has served as a useful framework for teacher training interventions (Dang, 2013) as well as analysing contradictions in teaching activity at the micro level (Nguyen & Nguyen, 2019; Nguyen, 2017). Within CHAT, there are two discernible traditions, one theorised by Leontiev (1981) and another later proposed by Engeström (1987, 2001), which are henceforth referred to as L-CHAT and E-CHAT respectively. While the former explores human psychology on the basis of Marxism, the latter was proposed in order to study organisational learning and change (Kaptelinin, 2005).

In previous studies, VST, L-CHAT and E-CHAT are often conflated, which may undermine the theoretical framework and create challenges in interpreting research findings (Kaptelinin, 2005; Martin & Peim, 2009). Kaptelinin (2005) contends “[t]he different meanings of the concept within these approaches may cause certain problems for researchers and practitioners” (p. 11). In addition, the confusion among these strands also leads to mixed labels for the theory, for example, Vygotskian activity theory perspective (Kim & Zhang, 2013), Vygotskian and cultural-historical theory perspective (Yang & Markauskaite, 2021) and the like.

Clarifying these traditions is thus an important and urgent task given the prevalence of these approaches in the field of education. This paper thus aims to bridge this gap. In doing so, it begins with an overview of these approaches, followed by a critical discussion of their similarities and differences. Finally, implications for research and practices are provided. This paper contributes to the literature an essential theoretical discussion which deepens the use of these sociocultural approaches, with insights valuable and applicable to various educational activities.

## Vygotsky’s Sociocultural Theory

VST owns its name to Lev Semyonovich Vygotsky, a Russian psychologist (1886–1934). The theory emerges through Vygotsky’s efforts to innovate the field of psychology in his time. Vygotsky and his contribution are “prized for having innovatively redefined the development of mind with the contingencies of history and socio-cultural context” (Martin & Peim, 2009, p. 132). While VST features psychological topics and investigations, prominent concepts frequently adopted in educational research involve *mediation*, *zone of proximal development*, *internalisation* and *perezhivanie*. These concepts are closely interrelated, constituting the system of VST. As Veresov (2016) puts it, a concept should be interpreted “by clarifying the place and role of this concept within cultural-historical theory and examining the connections of this concept with other concepts, principles, and laws of the theory” (p. 129). In the following subsections, these concepts are in turn briefly presented and where relevant, their interrelations are discussed.

## Theoretical concepts

### Mediation

The first concept, mediation, is concerned with the nature of human psychological processes, particularly higher mental functions. As such, mediation serves as a fundamental concept and principle in VST. The theory stipulates that human higher mental functions are mediated with signs, the “artificial, or self-generated, stimuli”, (Vygotsky, 1978, p. 39). Examples of signs are speech, written language, mathematical symbols and other sign systems. The use of signs alters the direct relation between humans and environment or between stimulus and response, turning it into a mediated relation. For instance, speech has been demonstrated as enhancing human problem-solving capacity. Vygotsky (1978) maintains that “the most significant moment in the course of intellectual development, which gives birth to the purely human forms of practical and abstract intelligence, occurs when speech and practical activity, two previously completely independent lines of development, converge” (p. 24).

Signs (e.g. language) play multiple psychological functions in supporting and regulating human activities. According to Vygotsky (1978):

The specifically human capacity for language enables children to provide for auxiliary tools in the solution of difficult tasks, to overcome impulsive action, to plan a solution to a problem prior to its execution, and to master their own behavior. (p. 28).

**Fig. 1:**
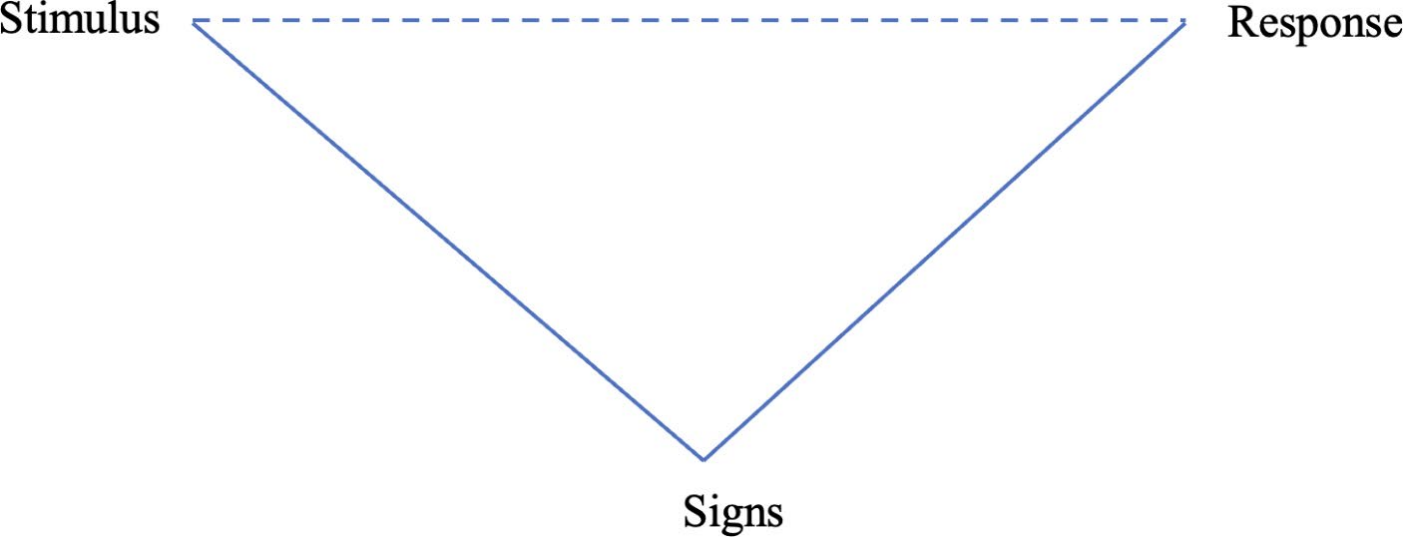
The role of Mediational Role of Signs in regulating human response, adapted from Vygotsky (1978)

Figure 1 illustrates the mediational role of signs in mediating an individual’s response to environmental stimulus. While in elementary functions, the relation between stimulus and response is a presupposed direct relation, with the mediation of signs, the relation becomes indirect. According to Vygotsky (1997), sign mediation is a fundamental feature of all higher mental functions. The incorporation of signs creates a new psychological structure, of which the sign is a part or a component (Toomela, 2016; Vygotsky, 1997). For this reason, some psychic operations are impossible without semiotic mediation.

Additionally, the value of signs lies in the fact that they possess “the specific function of reverse action”, which “transfers the psychological operation to higher and qualitatively new forms and permits humans, by the aid of extrinsic stimuli, *to control their behavior from the outside*” (Vygotsky, 1978, p. 41, emphasis in original). An example of the reverse function of signs is when a child can successfully remember a word by means of a picture. The fact that by looking at the picture the child can retrieve in his memory the word indicates the quality of reverse action.

Sign mediation thus allows for far freer and more complex psychic operations. Yet, mediation should not be conflated with development from VST perspective (Hakkarainen, 2004). In addition to semiotic mediation, it has also been empirically demonstrated that higher psychological operations may also require the development of logical/scientific concepts or advanced forms of the word-meaning structure (e.g. Toomela 2020; Toomela et al., 2020).

### Internalisation

The second principle deals with the process whereby an external activity (i.e. performed in social interactions) is gradually assimilated into the existing psychological system of the individual to allow for internal mental development. Internalisation is also referred to as the genetic law of cultural development, which states that:

[A]ny function in the child’s cultural development appears on stage twice, that is, on two planes. It firstly appears on the social plane and then on a psychological plane. Firstly it appears among people as an interpsychological category, and then within the child as an intrapsychological category. This is equally true with regard to voluntary attention, logical memory, the formation of concepts and the development of volition. (Vygotsky, 1983, p. 145, as cited in Veresov, 2014)

Internalisation involves a hierarchical structural reorganisation of the existing mental system to assimilate new cultural signs and operations (Fleer et al., 2017; Toomela, 1996; Vygotsky, 1997). Indeed, the historical development of higher mental functions is understood in VST “as the history of the transformation of means of social behaviour into means of individual psychological organization” (Vygotsky & Luria, 1994, p. 138). This genetic law of cultural development emphasises the significance of social interactions/relations in an individual’s psychological development, which highlights the social genesis of higher mental functions.

### Perezhivanie

The next concept, perezhivanie, concerns the differential influence of environment on individual development. Although popularised relatively recently compared to other VST concepts, perezhivanie has rapidly gained traction in the literature (Veresov, 2016, 2020; Wei, 2021; Yang & Markauskaite, 2021). The reading on the concept is however limited, and can be found mostly in the book chapters, “*The Problem of Environment”* (Vygotsky, 1994) and “*The Crisis at Age Seven”* (Vygotsky, 1998). The central idea behind the concept is that environment though being critical in one’s development cannot be equated with the latter per se. In other words, environment only provides the materials for development. Vygotsky maintains “for a proper understanding of the role which environment plays in child development it is always necessary, if one can put it this way, to approach environment not with an absolute but a *relative* [emphasis added] yardstick” (p. 338). As such, we cannot assume a deterministic and objective relation between environmental factors and a person’s internal development. The differential impact of environment is also due to the fact that a person’s experience is always coloured with personal meanings. As Vygotsky (1978) points out, “I do not see the world simply in color and shape but also as a world with sense and meaning” (p. 33).

Perezhivanie plays a fundamental role in moderating the influence of environment on the individual. As Vygotsky (1994) argues, “it is not any of the factors in themselves (if taken without reference to the child) which determines how they will influence the future course of his development, but the same factors refracted through the prism of the child’s perezhivanie” (p. 340). This is not to deny the effect of environmental factors for they indeed play a controlling role in the developmental process (Vygotsky, 1998). Perezhivanie serves as the *prism* that refracts the impact of environment (Veresov, 2016, 2020) and thus plays a crucial role in determining the development of the individual. Vygotsky (1994) contends that “the essential factors which explain the influence of environment on the psychological development of children, and on the development of their conscious personalities, are *made up of their perezhivanija*^1^ [emphasis added]” (p. 339). In studying perezhivanie, Vygotsky (1994) stipulates the need to “be capable of finding the particular prism through which the influence of the environment on the child is refracted, … in other words how a child becomes aware of, interprets, [and] emotionally relates to a certain event” (p. 341). Accordingly, an investigation into perezhivanie can be afforded by exploring how an individual understands the situation and associated emotions (e.g. Huh & Kim 2021; Yang & Markauskaite, 2021).

### Zone of Proximal Development

Finally, the last concept, also possibly the most widely adopted in educational research, is zone of proximal development (ZPD). Yet, despite its widespread adoption, the interpretation of this concept remains contestable and contradictory in contemporary literature (Kostogriz & Veresov, 2021). In general, Vygotsky (1978) differentiates between the actual level of development (ALD), which he regards as “the level of development of a child’s mental functions that has been established as a result of certain already *completed* developmental cycles” (p. 85, emphasis in original). On the other hand, ZPD refers to “the distance between the level of actual development, as identified with the help of the tasks the child solves independently, and the level of possible development, identified with the help of the tasks the child solves under the guidance of adults and in cooperation with more competent peers” (Vygotsky, 1935, p. 35, as cited in Kostogriz & Veresov 2021). People at the same biological age may differ in their ability to solve a problem with the support of adults. Using standardised tests, as Vygotsky sees it, is only adequate for examining the test-takers’ ALD. In plain terms, the difference between ALD and ZPD can be explained as independent problem-solving and problem-solving with others’ support.

However, it should be acknowledged that whether the concept of ZPD is central to VST is still subject to controversy. On the one hand, it is considered a central concept linked to the genetic law of psychological development, which can in turn have methodological implications for the modern psychology (Kostogriz & Veresov, 2021). On the other hand, it has also been argued that ZPD has been defined by Vygotsky differentially and is rather a descriptive and theoretically underdeveloped concept (cf. Toomela, 2015; Valsineer & Van der Veer, 1993).

### Integrating VST concepts

While the four concepts presented above are undoubtedly important, VST is non-reducible to a combination of these concepts. Toomela (2015) warns against such a reductionist approach for “it does not make up any theory of human mind but rather a list of some quite trivial ideas” (p. 319). Vygotsky understands and investigates the human mind as a “structural-systemic” phenomenon and a scientific analysis from VST perspective needs to be able to “reveal the elements from which the mind is composed, the specific relationships between the elements, and qualities of the whole that emerges in the synthesis of the elements” (p. 318). A proper interpretation and application of VST and its concepts must essentially reflect this structural-systemic epistemology.

**Fig. 2:**
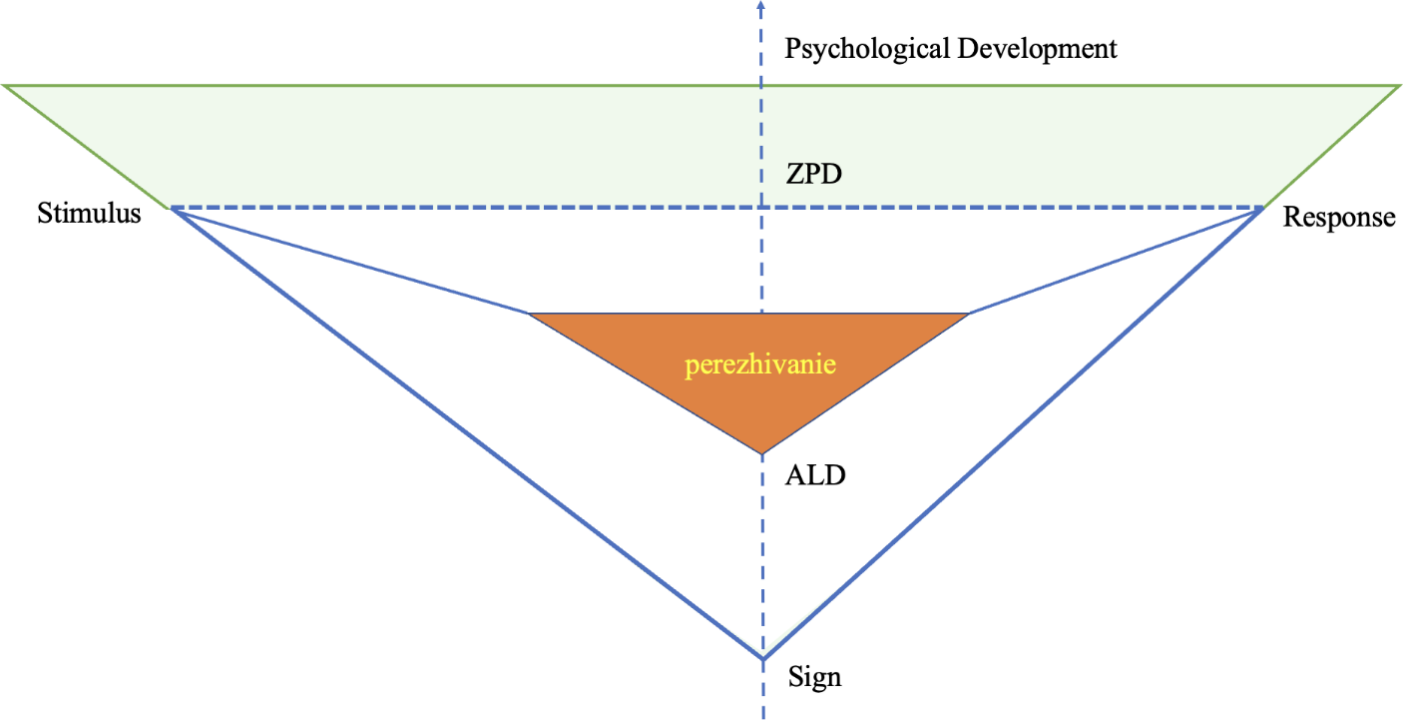
A conceptual model of VST theoretical concepts

To further account for the interrelations among the concepts discussed above, a conceptual model (see Fig. 2) is proposed. As indicated in the model, the relation between stimulus and response is mediated by signs, reflecting Vygotsky’s conceptual triangle of mediation (see Fig. 1). The direct line represents a direct relation, whereas the dotted line suggests an intermediate one. Perezhivanie is placed at the heart of the model, underscoring its fundamental role in refracting the environmental impact and ultimately determining the type of psychological development of the individual. The impact of the stimulus is refracted in the individual’s perezhivanie, which can lead to differential responses within and across individuals depending on the personal characteristics mobilised in the situation (Vygotsky, 1994, 1998). Additionally, the triangle with the white background indicates the ALD of an individual, whereas the light green represents their ZPD. Here development is presented as an upside-down pyramid, resonating with how Vygotsky understands concept formation, that is, “the pyramid of concepts is turned on its head” (Vygotsky, 1987, p. 128). In particular, development is understood as occurring in an expanding vertical manner: “Development, as often happens, proceeds here not in a circle but in a spiral, passing through the same point at each new revolution while advancing to a higher level” (Vygotsky, 1978, p. 56).

Although development is illustrated as a vertical line in the model, it should be interpreted more dynamically. From VST perspective, development should not be understood in evolutionary terms, i.e. as a linear process with changes gradually and increasingly accumulated. As Vygotsky argues, development occurs in a more complex manner, which essentially features non-linearity and complex qualitative reorganisations of the existing psychological system.

[I]t is a complex dialectical process that is characterized by complex periodicity, disproportion in the development of separate functions, metamorphoses or qualitative transformation of certain forms into others, a complex merging of the process of evolution and involution [emphasis added], a complex crossing of external and internal factors, a complex process of overcoming difficulties and adapting. (Vygotsky, 1997, p. 99)

In general, the four concepts offer important insights into VST and have been widely applied in the empirical literature. However, they must be interpreted and utilised in alignment with VST’s fundamental principles, for instance, its structural-systemic epistemology and qualitative reorganisation of the existing mental system. For instance, Chaiklin (2003) argues that it is crucial “to understand what Vygotsky meant by development in general, if we are going to understand what he meant by zone of proximal development in particular” (p. 46).

## Leontiev’s and Engeström’s Cultural-Historical Activity Theory

CHAT focuses on exploring the role of object-oriented activities on human development and social transformation. Within CHAT, two versions of the theory can be discerned, the original one established by Leontiev (1978, 1981) and the later versions proposed by Engeström Engeström (1987, 2001). The following subsections provide more information about these two traditions.

### Leontiev’s Cultural-Historical Activity Theory (L-CHAT)

L-CHAT has its origins in German philosophy and Russian school of psychology and is often associated with the works of Leontiev (1978, 1981). Yet, the concept of activity has its long tradition in the works of various Russian psychologists with Leontiev often being recognised as the most prominent theorist (see Chaiklin 2019). Within L-CHAT, object and activity are two fundamental concepts. Let us start with the concept of object. For Leontiev, object can be understood in both a broad and a narrow meaning. Leontiev (1981) explains:

Usually this concept has two meanings: in a broad sense, it is a thing related to other things, that is, a “thing having an existence;” in a more narrow sense, it is something that opposes (German *Gegenstand*), something that resists (Latin *objectum*), something at which an action is directed (Russian *predmet*). (p. 49)

The broad meaning of object is anything that has its own existence, which may or may not exist in a person’s consciousness (e.g. a foreign language, a country). In a narrow meaning, object is the entity (i.e. both mental or physical) that the individual’s activity is oriented toward, “something at which an action is directed”. Examples of object involve “the object of eating,” “the object of labor,” “the object of contemplation” (Leontiev, 1981, p. 49).

As for the concept of activity, activity refers to both mental and materialistic processes, i.e. “no matter if this activity is an external one or an internal one” (Leontiev, 1981, p. 49). An activity is always social: “activities [in L-CHAT] can be either individual or collective in respect to their form, but they are always social” (Kaptelinin, 2005, p. 9). It is also essential to point out the inherent relation between activity and object where the latter determines the structure of the former. Importantly, the process of carrying out an activity transforms both the object and the subject. In particular, engaging with the object helps the subject to understand it better, which in turn enhances their activity (Kaptelinin, 2014).

Methodologically, L-CHAT theorises three levels of analysis: activity underpinned by motive, action by conscious goals and operation by conditions. According to Leontiev (1978):

[L-CHAT’s] analysis isolates separate (specific) activities in the first place according to the criterion of motives that elicit them. Then actions are isolated—processes that are subordinated to conscious goals, and, finally, operations that directly depend on the conditions of attaining concrete goals. (pp. 66–67)

While motive directs an individual’s activity, concrete actions are oriented towards conscious goals. Operations refer to the method, i.e. how the individual perform the actions, and the conditions in that specific temporal setting. To put it simply, an individual carries a motive-oriented activity with multiple goal-oriented actions utilising situated operations.

In practice, there can seemingly be a disconnection between activity and actions due to the division of labour in society. Leontiev uses collective primeval hunting as an example. One member of the hunting group acts as a drum beater to scare the animal away. This action seems at first contradictory to the purpose of hunting. However, its purpose is actually to drive the animal toward a place where other hunters lie in wait. The action of the drum beater clearly has a role in realising the general activity of hunting. As such, it is essential for researchers to reveal the *true* object of an activity under investigation through scientific analysis: “Activity may seem objectless, but scientific investigation of activity necessarily requires discovering its object” (Leontiev, 1978, p. 52).

In addition to Leontiev, other influential Soviet L-CHAT theorists involve Davydov, Elkonin and Zankov, who have made a significant contribution to educational psychology. The works of these authors were more oriented towards the training/educational activity, particularly at primary and secondary levels. These scholars have capitalised on experimental research to provide empirical support for their frameworks (Guseva & Solomonovich, 2017; Matusov, 2001). In particular, Ekonin-Davydov’s and Zankov’s approaches represent two major pedagogical frameworks, also known as *developmental education* or *theory of developmental learning activity* (Matusov, 2001; Shadrikov & Kuznetsova, 2013). Major educational principles advocated feature an emphasis on general development, posing optimal difficulties to promote development, developing theoretical thinking and learning through engaging in practical activities. First, Zankov underscores the need to promote children’s general development, which involves holistically the mind, will and emotions:

General development, as well as comprehensive, is opposed to the one-sided, unilateral development. An analysis of the general development in psychological terms is done through certain forms of the mental activity. If we keep in mind the traditional division of the psyche into the mind, will and feelings, the general development includes all three of these lines. (Zankov, 1963, as cited in Guseva & Solomonovich, 2017, p. 777)

Second, another principle stipulates the importance of providing the learners with obstacles at an optimal difficulty for them to overcome because “[i]f the teaching material and methods of its learning are such that there is no obstacle for students to overcome, then the development of children is sluggish and weak” (Zankov, 1975, as cited in Guseva & Solomonovich 2017, p. 778). Third, theoretical concepts rather than empirical/everyday ones should be taught, particularly by cultivating the generalisation capacity of the learners:

Generalisation is the detection of the interrelationship between the general and the individual. The general contains the entire diversity of the individual. To make a generalization means to discover a principle, a necessary connection of the individual phenomena within a certain whole. (Davydov, 1990, p. 138)

Accordingly, in learning, children should be provided with the opportunities to observe, draw meaningful systemic connections and make conclusions about the phenomenon. Fourth, learning should be directed towards solving meaningful practical activities. For instance, instead of teaching mathematics by making children learn numbers as in traditional school, their mathematical learning should be oriented towards solving practical measurement tasks.

These principles have been successfully utilised to develop innovative curriculums applied in Russian public primary schools since the 1950s (Guseva & Solomonovich, 2017) as well as in mathematics programs in other countries such as Norway and Canada (e.g. Arginskaya et al., 2014; Arginskaya et al., 2001). For instance, Davydov’s didactic principles continue to be influential in studies examining innovative ways of teaching mathematics to young learners (e.g. Sidneva 2020; Venkat et al., 2021).

### Engeström’s Cultural-Historical Activity Theory (E-CHAT)

#### Generations of E-CHAT

The second variant of CHAT, E-CHAT, is attributed to the works of Engeström (1987, 1999, 2001). E-CHAT can be considered an outcome of Engeström’s efforts to re-interpret and re-theorize L-CHAT for studying organisational change (see Bakhurst 2009; Kaptelinin, 2005). Compared to L-CHAT, E-CHAT features an interdisciplinary theoretical basis, involving L-CHAT and many other theoretical and philosophical traditions (Kaptelinin, 2005). An activity is always understood in E-CHAT as being collective with its object being shared among the activity participants. Engeström (1987, 1999, 2001) has a large role in re-defining, theorising and illustrating generations of E-CHAT as models of activity systems.

Engeström & Sannino (2021) discuss four versions of E-CHAT. The first starts with Vygotsky’s concept of mediation. The second generation is illustrated as an activity system (see Fig. 2) with seven components, namely subject, mediational tools, object, rules, community, divisions of labour and outcomes.

**Fig. 3:**
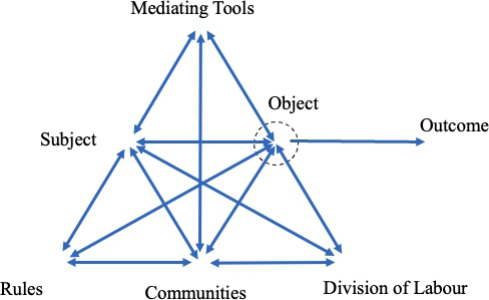
Second generation of E-CHAT, adapted from Engeström (2001)

Subject refers to the individuals who perform the activity, whereas object refers to the shared motive of the activity. Mediational tools can be physical and psychological. For example, in teaching activity, teachers’ psychological tools can involve pedagogies and concepts and the physical tools can be classroom, chalk and projector. Rules are concerned with the norms and regulations constraining or enabling the performance of the activity. Community indicates other groups of people involved in the activity. Examples of teacher communities are students, colleagues and their institutional administrators. Division of labour refers to how responsibilities are shared among members of the community.

**Fig. 4:**
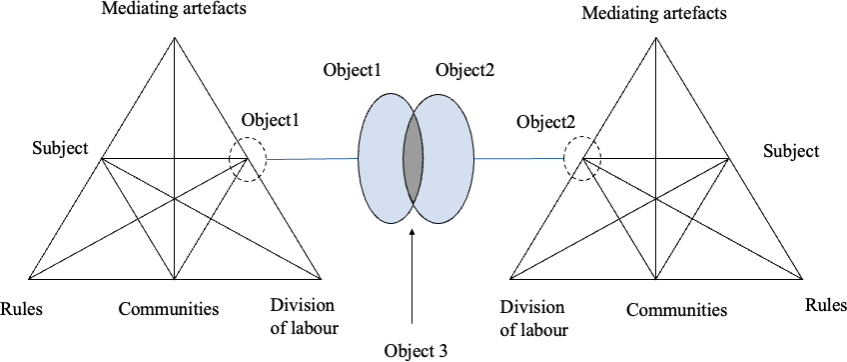
The third generation of E-CHAT, adapted from Engeström (2001)

As E-CHAT is widely adopted in many international contexts, the third generation (see Fig. 4) has been developed to provide “conceptual tools to understand dialogue, multiple perspectives, and networks of interacting activity systems” (Engeström, 2001, p. 135). The standard activity system is now modified to “include minimally two interacting activity systems” with a collectively shared object (Engeström, 2001, p. 136).

Finally, the fourth generation of activity deals with *runaway* objects or “critical societal problems” such as climate change and pandemics, which an interventionist approach (as in the third generation) cannot resolve (Engeström & Sannino, 2021, p. 6). As such, in this new generation, adding more activity systems to the unit is no longer considered appropriate or effective for data analysis. Instead, the focus must be on “the multiple coalescing cycles of expansive learning involved within and across the activities involved, their relatively independent dynamics and their interdependency” (Engeström & Sannino, 2021, p. 15). Nevertheless, the fourth generation is still in its nascent stage where “[w]e are only beginning to develop conceptual tools and methods to analyse and foster these processes of generating dynamic social cohesion around a shared object among heterogeneous activities” (Engeström & Sannino, 2021, p. 19).

#### Principles

Engeström (2001) summarises five principles underpinning E-CHAT: an activity system as the unit of analysis, the multiplicity of voice, historicity of activity system, contradiction as the developmental force, and transformation of the activity system. The first principle postulates “a collective, artifact-mediated and object-oriented activity system, seen in its network relations to other activity systems” as the prime unit of analysis (Engeström, 2001, p. 136). The second principle, concerning the multi-voicedness of activity systems, points out the multiplicity of viewpoints and histories of participants in the activity system constitutes the “source of trouble and innovation” (Engeström, 2001, p. 136). In the third tenet, the analysis of an activity system must be conducted taking into account its historicity of development, i.e. “the local history of the activity and its objects, and as history of the theoretical ideas and tools” (Engeström, 2001, p. 137).

The fourth principle stipulates that systemic contradictions serve as the inner force for change and development (Ilyenkov, 1977, 2008). Contradictions are defined as “historically accumulating structural tensions within and between activity systems”, irreducible to surface problems or conflicts (Engeström, 2001, p. 137). Tensions and conflicts are only surface manifestations of the underlying contradictions (Engeström & Sannino, 2011). By studying these observable tensions, we can then determine the system contradictions underpinning the activity systems.

Finally, the fifth tenet of E-CHAT is concerned with the transformation of an activity system, postulated as a collective effort through the ZPD, reconceptualized by Engeström (1987) as “the distance between the present everyday actions of the individuals and the historically new form of the societal activity that can be collectively generated as a solution to the double bind potentially embedded in the everyday actions” (p. 174). The inner force for the development of activity systems are contradictions, which then trigger the subject’s self-questioning and collective change effort, which ultimately results in the re-conceptualisation and expansion of the object and the motive of the activity to embrace significantly more possibilities (Engeström, 2001).

## Comparisons among VST, L-CHAT and E-CHAT

In this section, similarities and discernible features among VST, L-CHAT and E-CHAT are examined. However, such a comparison is essentially limited in scope, focusing on major features rather than a systematic or complete comparison among them, an extremely challenging given the long historical development of these traditions (see also Kaptelinin 2005). A more detailed comparison between E-CHAT and L-CHAT can be found in Cong-Lem (2022).

### Theoretical foundations

It is a commonly accepted fact that the Soviet cultural-historical school of psychology is heavily influenced by Marxism. However, the extent to which these cultural-historical traditions adopt the theory is debatable. First, for VST, although Vygotsky acknowledges the influence of dialectical materialism in his works, he stipulates the need for psychology to create its own *Capital* (Veresov, 2010). Vygotsky argues that dialectical materialism must be *translated* into the domain of psychology rather than be straightforwardly adopted:

*immediate application of the theory of dialectical materialism* [emphasis added] to the problems of natural science, in particular to biology and psychology, *is impossible* [emphasis added] as it is impossible to apply it immediately to history or sociology. ... [A] theory of ... psychological materialism, as a mediating science *explicating the concrete application of the abstract tenets of dialectical materialism* [emphasis added] to a particular domain of phenomena is indispensable.” (Vygotsky, 1982, pp. 419–420, as cited in Veresov, 2010)

For Vygotsky, psychology, as also true for other sciences, needs to create its own *Capital* in applying dialectical materialism.

In order to create such enabling theories - methodologies in general sciences - it is necessary to discover the essence of the given area of phenomena, the laws of their changes, their qualitative and quantitative characteristics, their causality; to create the categories and concepts relevant to them - in other words, to create one’s own *Capital*. (Vygotsky, 1982, p. 420, as cited in Veresov, 2010)

A more comprehensive historical analysis of the relationship between VST and Marxism can be found in Veresov (2010). In comparison to VST, L-CHAT seems loyal to and is largely established on the basis of Marxism. Leontiev (1978) highlights,

Soviet scientists countered methodological pluralism with *a unified Marxist-Leninist methodology* [emphasis added] that allowed a penetration into the real nature of the psyche, the consciousness of man. A persistent search for resolutions of the principal theoretical problems of psychology *on the basis of Marxism* [emphasis added] began. (p. 3)

As indicated in the citation, L-CHAT underscores the need to capitalise on Marxism in its methodology and “resolutions of the principal theoretical problems” of psychology. Finally, E-CHAT, claiming its origins in VST and L-CHAT (Engeström, 2001), is a multidisciplinary theory rooted in various anthropological, biological and philosophical traditions (Bakhurst, 2009; Kaptelinin, 2005). Engeström (1999) emphasises the interdisciplinary nature of E-CHAT:

Today activity theory is transcending its own origins: It is becoming truly international and multidisciplinary. This process entails the discovery of new and old related approaches, discussion partners, and allies, from American pragmatism and Wittgenstein to ethnomethodology and theories of self-organising systems .... ... I anticipate that the current expansive reconstruction of activity theory will actually lead to *a new type of theory* [emphasis added]. (p. 20)

As such, while L-CHAT aims to develop a psychological theory on the basis of Marxism, E-CHAT is open and has drawn on different theoretical strands. In other words, E-CHAT can be considered a product of the integration of both Russian and Western theoretical traditions.

### Domain-specific approaches

On the one hand, VST and L-CHAT are psychological theories and as such their focus is on explaining human psychology, which involves consciousness, personality and psychological processes (e.g. memory, cognition, emotion). The works of Vygotsky, for instance, can be separated into two major periods. His works in the first phase (1928–1931) focus on examining higher psychological functions, whereas in the second (1931–1934), his research focus shifted to studying human consciousness and its dynamic structures (Veresov & Mok, 2018). In a similar vein, the central phenomena under investigation in L-CHAT involve “the problems of activity, consciousness, and personality” as introduced by Leontiev in his seminal book (Leontiev, 1978, p. 9).

On the other hand, E-CHAT is later proposed by Engeström drawing on a multidisciplinary approach. As a scholar working in the field of adult education, Engeström interpreted and further developed CHAT to examine (informal) professional learning and organisational change. For example, E-CHAT has been utilised as a productive analytical framework to study preservice teachers’ professional learning (e.g. Dang 2013; Nguyen, 2017), in-service teachers’ professional development (Yamagata-Lynch & Haudenschild, 2009; Yan & Yang, 2019) and educational technology implementation (e.g. Demiraslan & Usluel 2008; Marwan & Sweeney, 2019). However, some scholars also note that the difficulty of E-CHAT lies in the fact that its activity systems are often considered rigid and inadequate to study complex psychological processes (Edwards, 2005; Toomela, 2000, 2008).

### Methodological approaches

VST and CHAT traditions also differ in their pursuit of theory and knowledge validation, which is largely due to their differential epistemological stances. VST adopts what is called *structural-systemic epistemology*, which seeks to reveal the structure of human psychological functions and the dynamic relationships among their elements (Toomela, 2000, 2008, 2015). Accordingly, his knowledge validation draws on experimental-genetic methodology, which is “a methodology of the experimental study of the very process of development, i.e. artificial reconstruction of the process from the very beginning, from the ‘bud’ of development to its ‘fruit’” (Veresov, 2014, p. 88). For instance, when investigating the difference in choice-making between children and adults, Vygotsky (1978) reports on his experiment requesting “four- and five-year-old children to press one of the five keys on a keyboard as they identified each one of a series of picture stimuli assigned to each key” (p. 33). His conclusion from the experiment is that “the entire process of selection by a child is *external*, and concentrated in the motor sphere, thus allowing the experimenter to observe the very nature of the choice process itself in the child’s movement” (p. 34, emphasis in original). In other words, the arguments in VST are not merely philosophical but are testable and replicable.

L-CHAT and E-CHAT have been argued to be more philosophical in nature (Bakhurst, 2009; Kaptelinin, 2005). According to Martin & Peim (2009), the fact that CHAT draws on Ilyenkov’s work makes it “heavily dependent on ‘speculative anthropology’ and that its attempt to produce an [sic.] historically sound and grounded ‘anthropogenesis’ is founded in the very assumptions it seeks to verify” (p. 132). This statement is more suitable to describe E-CHAT, which centres around the notion of *contradictions* (Ilyenkov, 1977, 1982). In addition, the three specific types of data Engeström (1987) utilised to validate his theory involve: (1) “theories and theoretical prepositions pertaining to human learning”, (2) “general historical accounts” and (3) literary works (e.g. *The Adventure of Huckleberry Finn* by Mark Twain) and historical events (pp. 11–12).

In the case of L-CHAT, the critique above appears to be only partially true and more appropriately describes the two books of Leontiev (1978, 1981) that were translated into English rather than his work in general. More experimental findings from the L-CHAT tradition can be found in the works of Leontiev’s disciples (e.g. Galperin, Stolin, Davydov).

### Concept of activity

The next point of comparison is concerned with *activity*, a central concept in L-CHAT and E-CHAT. It is not uncommon for modern scholars to attribute CHAT to Vygotsky’s works. However, other scholars have pointed out that this attribution seemed unlikely to be the case (see Havnes 2010; Lektorsky, 2009; Martins, 2013). Valsineer & Van der Veer (1993) maintain that “the representation of Vygotsky as one of the originators of Soviet ‘activity theory’ constitutes a historically recent exaggeration of the realities in Soviet psychology in early 1930s” (p. 51). Lektorsky (2009) reiterate this point:

Many scholars think that it is possible to consider Vygotsky’s conception as the first variant of cultural-historical activity theory. But Vygotsky himself did not speak about activity theory. Moreover, some of his pupils (A. N. Leont’ev, P. I. Zinchenko, and P. J. Gal’perin) and other psychologists (S. L. Rubinstein) criticized him for not taking into account the role of practical activity in the process of mediation. (p. 77)

This is not to say that the concept of activity has no important role in VST. However, CHAT traditions seem to overemphasise it in their theoretical frameworks leaving other fundamental tenets of VST unaddressed. Toomela (2000, 2008, 2016) points out, the systemic-structural principle in VST is largely missing in CHAT strands, resulting in the latter viewing psychic development in terms of linear causality.

If, however, the systemic nature of HPFs [Higher Psychological Functions] is ignored or denied, what is left over is the theory of linear causality. And the relationship between social world and individual mind becomes treated as linear social = cause → changing individual = effect way. Exactly this happened in so-called activity theory, founded by Leontiev (1981) and followed by many today (Toomela, 2016, p. 102).

Thus, the following comparison of activity in this paper is thus concerned primarily with CHAT approaches. L-CHAT theorises activity as an explanatory principle. It is the activity that mediates and transforms the object, the subject themselves (during the process) and the relation between them (Hakkarainen, 2004). On the other hand, in E-CHAT, activity refers to the central activity under investigation, for instance, teaching, research or professional development. This general activity is then conceptualised as an activity system with a minimum of six components as shown in Fig. 3. The major task of E-CHAT scholars is to investigate the contradictions within and between activity systems and their manifestations (Engeström & Sannino, 2011).

Next, another gap between the two CHAT approaches concerns the mode of activity. L-CHAT conceptualises activity as existing in both individual and collective modes. Leontiev (1978) emphasises that “[u]nder whatever kind of conditions and forms of human activity takes place, whatever kind of structures it assumes, it must not be considered as isolated from social relations, from the life of society” (p. 51). Davydov (1999) reiterates this point, contending “[a]ctivity exists in both collective and individual forms when a person acts as a generic social being” (p. 41).

On the other hand, E-CHAT provides an understanding of activity in a strictly collective sense. According to Engeström & Miettinen (1999),

Mediation by other human beings and social relations was not theoretically integrated into the triangular model of action [in Vygotsky (1978)]. Such an integration required a breakthrough to the concept of activity by distinguishing between collective activity and individual action. This step was achieved by Leont’ev by means of reconstructing the emergence of division of labor. (p. 7)

As such, the discernible difference in understanding the mode of activity is highlighted by Kaptelinin (2005): “The … distinction between collective activities and individual actions [of E-CHAT] is not consistent with the general framework developed by Leontiev” (pp. 11–12). This is possibly due to Engeström’s misinterpretation of Leontiev’s intention when discussing the collective hunting example. The example is meant to illustrate a seemingly disintegrated connection between the general motive (i.e. hunting) at the activity level and what an individual member does at the action level (i.e., scaring the animal away toward the area ambushed by other members) rather than to define activities as strictly collective practices (see also Kaptelinin 2005).

## Implications

By exploring the central tenets of these cultural-historical traditions, we are in a better position to utilise them to serve our research and educational purposes. In this section, I outline several suggestions for utilising these frameworks to support educational purposes.

First, E-CHAT offers an interventionist framework to support professional learning and development. For instance, the third generation has been productively utilised to support preservice teacher learning in paired placement (e.g. Dang 2013). Also, E-CHAT can be used to frame and study the contradictions and tensions in teaching activity (e.g. Barahona 2015; Karimi & Mofidi, 2019; Kim, 2011; Nguyen, 2014, 2017), teacher identity development (e.g. Karimi & Mofidi 2019) and teacher agency (e.g. Kitade 2015; Yang, 2012).

On the other hand, various concepts of VST have also been explored and further developed. Johnson & Golombek (2016) theorised the concept of *responsive mediation* based on Vygotsky’s works. They also explored the applications of other concepts such as perezhivanie, obuchenie and ZPD, which supported professional development and dynamic assessment of second language teachers. In recent years, perezhivanie, broadly explored in terms of how an individual interprets and relates emotionally to a professional situation, has also become a prominent concept utilised to study teachers’ subjectivity, identity, agency and professional development (e.g. Dang 2013; Golombek, 2015; Golombek & Doran, 2014; Huh & Kim, 2021; Wei, 2021).

Additionally, previous scholars have also become mindful of the domain-specific nature of these cultural-historical traditions and called for their integration to better address complex educational issues. As a psychological theory, VST focuses on studying individual mental processes. L-CHAT and E-CHAT can be considered extensions of VST which are “custom designed to deal successfully with practical and research issues in their respective domains, that is, psychology and organisational change” (Kaptelinin, 2005, p. 11). While VST offers powerful theoretical lenses through which individual consciousness and subjectivity can be examined (Edwards, 2005; Stetsenko, 2005), E-CHAT offers an analytical framework to analyse tensions and transformations of collective professional activities. In order to draw on the strengths of these cultural-historical traditions, previous researchers have integrated them into a holistic framework (e.g. Dang 2013; Feryok, 2012; Lantolf & Pavlenko, 2001).

Nevertheless, given the differential epistemological stances between these theories (Toomela, 2000, 2008, 2015), the question whether and, if yes, to which extent these cultural-historical traditions can be integrated should be further theoretically and empirically investigated. E-CHAT as a general analytical framework has often been critiqued for its inadequacy in extrapolating subjectivity and individual contribution in change and development (Edwards, 2007; Eteläpelto et al., 2013; Yang, 2012) as well as in studying psychological processes (Toomela, 2000, 2008). Importantly, it seems more fruitful and theoretically plausible to utilise VST concepts to complement CHAT rather than doing the reverse. For example, Yang (2012) combined E-CHAT and ZPD (from VST) to explore Chinese EFL teachers’ professional agency, where the former served as an analytical framework to uncover systemic contradictions and the latter to identify the levels of teacher agency development. In the same vein, Dang (2013) utilised E-CHAT, ZPD and perezhivanie to establish a unifying framework to study how Vietnamese preservice teachers responded differentially to their co-teaching program and thus experienced identity development accordingly.

## Conclusions

The current paper summarises three cultural-historical traditions, VST, L-CHAT and E-CHAT, which are confused and conflated. Due to their underlying theoretical differences, they should be recognised as embedded strands within the overarching cultural-historical tradition, as established by VST. These theoretical approaches have also been compared to unveil their differences in theoretical orientations, which are often hidden and ignored in the literature. In addition to drawing on specific concepts from these theories, integrating them to establish a unifying theoretical framework is increasingly recognised as a viable approach to empowering educational research and practices.

Several limitations of the current paper should be mentioned. First, the ideas or arguments presented in this paper though drawing on the views of prominent scholars in the field, are undoubtedly debatable. For instance, whether Vygotsky’s theory is faithful to Marxism is still a complex and controversial issue. Second, due to the blurred boundaries among these traditions, previous scholars have often been found to draw upon more than one cultural-historical traditions within or across their works. Accordingly, the classification of previous empirical literature into VST, L-CHAT or E-CHAT is tentative and primarily based on the author’s judgement of the central framework underpinning the involved studies. Further theoretical discussion/development and empirical research are warranted to establish a more granular understanding of these traditions and to advance the field forward.

## Data Availability

The author does not analyse or generate any datasets, because our work proceeds within a theoretical approach.

## Footnotes

1 perezhivanija is the plural form of the noun perezhivanie.
